# Cephalexin Induced Cholestatic Jaundice

**DOI:** 10.1155/2014/260743

**Published:** 2014-08-07

**Authors:** Abhinav Agrawal, Mana Rao, Sarfaraz Jasdanwala, Ajay Mathur, Margaret Eng

**Affiliations:** ^1^Department of Internal Medicine, Monmouth Medical Center, Long Branch, NJ 07740, USA; ^2^Section of Infectious Diseases, Monmouth Medical Center, Long Branch, NJ 07740, USA

## Abstract

Cephalexin is a very commonly prescribed orally administered antibiotic which has many potential side effects. Amongst these cholestatic jaundice has been infrequently reported as an adverse reaction. We present a case of a 57-year-old male who exhibited features of cholestatic jaundice including elevated liver function tests (LFTs) after taking cephalexin and showed improvement on removal of the offending agent. During this time he was symptomatically treated with cholestyramine. Complete resolution of LFTs was seen in four weeks. Cephalexin induced cholestasis is rare and hence requires a high degree of clinical suspicion for prompt diagnosis and treatment.

## 1. Case Presentation 

57-year-old male presented to the emergency department with shortness of breath, leg cramps, and a skin rash 6 days after being started on cephalexin for a neck abscess. The patient also complained of generalized pruritus. His past medical history was significant for diabetes and the medications he was taking for it were metformin and glipizide. He had no history of alcohol abuse. On physical examination his vitals were normal. A maculopapular rash was noted on his trunk, back, buttocks, and forearms. He also had scleral icterus. The remainder of his physical exam including the abdominal exam was normal.

On the day of admission laboratory findings were Aspartate transaminase−268 u/L (8–48), Alanine transaminase (ALT)—267 u/L (7–55), alkaline phosphatase—429 u/L (45–115), total bilirubin—4.5 mg/dL (0.1–1.0), and direct bilirubin—3.5 mg/dL. Markers for synthetic function of the liver including albumin, prothrombin time, and thyroid function tests were all normal. Subsequently tests for acute viral hepatitis A, B, and C, CMV, HSV, and EBV were negative. Tests for metabolic liver diseases such as hemochromatosis, Wilson's disease, and alpha-1 antitrypsin deficiency were also negative. He was worked up for other possible causes of obstructive jaundice such as primary biliary cirrhosis and sclerosing cholangitis with anti-nuclear, anti-mitochondrial, and anti-smooth muscle antibodies, all of which were also negative. CT of the abdomen showed a normal sized liver and spleen along with cholelithiasis without any evidence of biliary duct dilatation. Ultrasound of the abdomen showed cholelithiasis without any other significant findings.

Cephalexin was immediately stopped. The patient was also put on cholestyramine with symptomatic relief of the itching. The patient was also put on prednisone with the dose being tapered. Over the following 2 days, his liver function tests (LFTs) trended down slightly and the skin eruptions resolved. The patient was discharged home with prescriptions of oral doxycycline for the abscess; cholestyramine and prednisone taper. The patient was followed up as an outpatient after 4 weeks, at which point his LFTs were noted to be completely normal.

## 2. Discussion

Cephalosporins are B-lactam antibiotics that inhibit bacterial peptidoglycan cell wall. They are classified into generations based upon the spectrum of antimicrobial activity. Cephalexin is a first generation cephalosporin available for oral administration. Hypersensitivity is the most common overall side effect of cephalosporins. With cephalexin, most common side effects include nausea, vomiting, and gastrointestinal (GI) disturbances [1].

Drug-induced liver injury (DILI) has an estimated annual incidence between 10 and 15 per 10,000 to 100,000 persons exposed to prescription medications. Many patients with DILI are asymptomatic and are only detected because of laboratory testing. Patients with acute DILI who are symptomatic may report malaise, low-grade fever, anorexia, nausea, vomiting, right upper quadrant pain, jaundice, acholic stools, or dark urine. Patients with DILI may also have signs and symptoms of a hypersensitivity reaction, such as a fever and rash [2]. DILI based on clinical presentation can be hepatocellular, cholestatic, or mixed. Five patterns of drug-induced cholestasis have been described [3]; each has its own characteristic biochemical, histological, and clinical manifestation. Drug-induced cholestasis most often manifests histologically in a hepatocanalicular cholestatic hepatitis pattern. Elevated transaminases have been reported with cephalexin use. Hepatitis has been reported with other cephalosporins including cefdinir and cefazolin [4, 5]. Cholestatic jaundice has been rarely reported with cephalosporins. One of the hypotheses included hypersensitivity in view of similar reactions induced by structurally related penicillins [6].

From an electronic database in the United States of America over the past 10 years, 7297 people self-reported side effects with cephalexin. Of these, only 2 patients (0.03%) reported having jaundice [7]. Our patient was treated with cephalexin for an ulcer on the back of his neck and had finished 6 days of therapy. Extensive history taking, laboratory testing, and diagnostic imaging for alternative explanations of the patients' symptoms were negative. In addition, the patient presented with rash along with jaundice another fact that supports an underlying immunological phenomenon and DILI. To the best of our knowledge there have been only 2 reported cases of cephalexin causing cholestatic jaundice. Skoog et al. [8] reported a 51-year-old male who received cephalexin for 10 days and developed cholestatic jaundice secondary to the drug. Singla et al. [9] reported a 21-year-old female who received cephalexin for 10 days after mammoplasty and 4 weeks later developed jaundice. The case reported by Skoog et al. had much higher level of bilirubin elevation (17.9) and thus took 7 weeks for complete resolution of the symptoms and enzyme levels. In comparison, this case was more similar to the case reported by Singla et al., where the highest bilirubin level was 4.3 and resolution was attained in 4 weeks.

A number of scales have been developed that attempt to codify causality of drug toxicity into objective criteria [10]. International criteria for liver toxicity were established by the Council of International Organizations of Medical Sciences (CIOMS). Evaluations should always include hepatitis and autoimmune serologies and appropriate imaging studies [11]. The Naranjo Adverse Reactions Probability Scale (NADRPS) also has been used in the past. In our patient the scores and probability based upon this scale were as follows: (1) Naranjo algorithm score—6, indicating causality as “probable ADR,” and (2) CIOMS/RUCAM score—6, indicating causality as “probable.” None of these scales are all encompassing and thus are not routinely used in the clinical setting. Thus, based upon the above mentioned scales, cephalexin was a probable cause of cholestatic jaundice in our patient. Rechallenge was not done due to inherent risk involved but the patient showed positive response to the removal of offending agent.

It is important to note that the risk of cholestatic jaundice and hepatic toxicity is higher in patient's taking multiple medications because of their potential to cause DILI. In our case, the patient was also taking metformin. A review of the literature has shown metformin to cause jaundice, either cholestatic or hepatocellular. Although metformin is not metabolized in the liver, the possible mechanisms of injury are either direct, idiosyncratic, or a drug-drug interaction [12, 13]. The usual duration of onset of presentation ranges from 1 to 4 weeks based on the reported cases. [12–14]. In our case, the patient had been taking metformin for >10 years duration and the improvement in symptoms occurred without the cessation of metformin. Thus, metformin as a cause of cholestatic jaundice was considered unlikely in our patient.

Mild pruritus can often be managed by nonspecific measures such as emollients and warm baths and/or histamine 1-receptor blockers such as hydroxyzine and diphenhydramine due to their sedative properties. Bile acid resins (cholestyramine or colestipol) are the first line agents in moderate to severe pruritus particularly when associated with excoriations and disturbed sleep. Our patient was started on cholestyramine with symptom improvement. Fat soluble vitamins (A, D, and K) should be replaced via the parenteral route in patients with long standing cholestasis [9]. In our patient the liver enzymes trended down relatively quickly after stopping the cephalexin and hence this was not required.

It is important to realize that although uncommon, cholestatic jaundice is one of the side effects of cephalexin. Clinicians should keep in mind this potentially serious side effect of severe hepatotoxicity and maintain a high degree of suspicion in order to promptly remove the offending agent in similar clinical presentations.
